# Regulation of cytoplasmic polyadenylation can generate a bistable switch

**DOI:** 10.1186/1752-0509-6-12

**Published:** 2012-02-15

**Authors:** Naveed Aslam, Harel Z Shouval

**Affiliations:** 1 Department of Neurobiology and Anatomy, The University of Texas, Medical School, 6431 Fannin Street, Houston, Texas 77030, USA

## Abstract

**Background:**

Translation efficiency of certain mRNAs can be regulated through a cytoplasmic polyadenylation process at the pre-initiation phase. A translational regulator controls the polyadenylation process and this regulation depends on its posttranslational modifications e.g., phosphorylation. The cytoplasmic polyadenylation binding protein (CPEB1) is one such translational regulator, which regulates the translation of some mRNAs by binding to the cytoplasmic polyadenylation element (CPE). The cytoplasmic polyadenylation process can be turned on or off by the phosphorylation or dephosphorylation state of CPEB1. A specific example could be the regulation of Calcium/Calmodulin-dependent protein kinase II (αCaMKII) translation through the phosphorylation/dephosphorylation cycle of CPEB1.

**Result:**

Here, we show that CPEB1 mediated polyadenylation of αCaMKII mRNA can result in a bistable switching mechanism. The switch for regulating the polyadenylation is based on a two state model of αCaMKII and its interaction with CPEB1. Based on elementary biochemical kinetics a high dimensional system of non-linear ordinary differential equations can describe the dynamic characteristics of the polyadenylation loop. Here, we simplified this high-dimensional system into approximate lower dimension system that can provide the understanding of dynamics and fixed points of original system. These simplified equations can be used to develop analytical bifurcation diagrams without the use of complex numerical tracking algorithm, and can further give us intuition about the parameter dependence of bistability in this system.

**Conclusion:**

This study provides a systematic method to simplify, approximate and analyze a translation/activation based positive feedback loop. This work shows how to extract low dimensional systems that can be used to obtain analytical solutions for the fixed points of the system and to describe the dynamics of the system. The methods used here have general applicability to the formulation and analysis of many molecular networks.

## Background

Cellular signaling pathways that can operate in a switch like manner are called bistable systems [1,2]. A bistable system has the ability to switch between two distinct stable steady states and such a system cannot rest in any intermediate state [3,4]. In response, to an external stimulus a bistable system can move from one state to another. If this switching is permanent then such a system is called irreversible otherwise it is a reversible switch [5]. The bistability in a signaling network is typically due to a positive feedback loop or double negative feedback loop [3]. However, the presence of a positive or a double negative feedback loop does not guarantee bistability [4]. In addition, to these feedback loops a biological network must have non-linear interactions to exhibit a bistable behavior. Previous, experimental work has described several examples of naturally occurring bistable system [6-18]. Still bistability is not considered to be a unifying theme of cellular signaling networks and more experimental work is needed to establish bistability as one of the general mechanism of cell signaling [4]. Typically, bistable biological systems were described either at the level of gene expression due to the regulation of gene expression by transcription factors, or at the level of posttranslational modifications e.g., activation-deactivation cycle due to phosphorylation. Here, we present a model of bistability that can arise from the control of gene expression at the level of translation of new proteins

Cytoplasmic polyadenylation regulates the translation efficiency of certain mRNAs through modulating the length of 3' poly (A) tail [19,20]. Polyadenylation can be regulated through a short nucleotide sequence known as cytoplasmic polyadenylation element (CPE) located in their 3' UTR. A CPE binding protein (CPEB1), represses the translation through its dual interactions with CPEs and other mRNA binding proteins. Phosphorylation of CPEB1 changes its interactions with these other proteins, promotes polyadenylation and increases the length of the poly (A) tail [19,20]. The mRNAs with a longer poly (A) tail are more likely to be translated compared to mRNAs with a shorter poly (A) tail [21,22]. The exact mechanism through which the longer poly (A) tail enhances the translation efficiency is not clear. However, it is believed that longer poly(A) tail leads to a circular mRNA which enhances the translation efficiency through recycling the translation machinery on the mRNA frame thus increasing the possibility of mRNA translation through initiation[23,24].

The translation of a highly abundant brain protein αCaMKII (2% of total brain protein is αCaMKII) is regulated through activity induced polyadenylation. The αCaMKII-mRNA contains two CPE elements in its 3'UTR and its translation can be regulated through phosphorylation of CPEB1 [25,26]. Recent studies have shown that αCaMKII can phosphorylate CPEB1 and therefore, possibly modulate its own translation through a positive feedback loop. Here, we examine the hypothesis that the positive feedback loop between αCaMKII and CPEB1 forms a bistable switch which regulates the translation of αCaMKII. The aim of this paper is to obtain analytical expressions for the bistability of the CPEB1- αCaMKII molecular pair as a generic example for such systems.

In this paper, we analyze the mathematical properties of this molecular loop. The characteristics of this loop are analyzed by evaluating the dynamics and directly locating the fixed points. Using the elementary biochemical kinetics we develop a molecular model of self-sustained polyadenylation based translation loop. This simple molecular model is represented by six differential equations. We simplify this model through introducing an approximation and algebraic manipulations. These, systematic simplifications result in a three dimensional differential equation based model. Further approximations can reduce this to a single dynamical equation. Based on our approximate equation we also developed an approximate analytical method to directly locate the fixed points of this system. We compare these analytical results to the numerical bifurcation diagrams obtained through numerical tracking of the complete system of equations and show their correspondence. Our results demonstrate that such a positive feedback loop which involves the control of translation through polyadenylation can indeed be bistable over a wide range of parameters. This simplified model, though motivated by the αCaMKII-CPEB1 loop, could be seen as a generic model for such a positive feedback loops which involves translation, and degradation of proteins. Such feedback loops do not conserve the quantity of these proteins and are therefore qualitatively different than most post translational feedback models [3,4]. Since, we use a simplified model we can obtain approximate analytical results, which provide us with intuition about how such feedback systems operate.

## Method

### A. Complete Model Equations

The following set of reactions is used to describe the interactions between the αCaMKII and CPEB1 molecule. These biochemical reactions are based on elementary Michalis-Menten type kinetics. The dynamical variable X represents αCaMKII and Y represents the CPEB1. The P subscript represents the phosphorylated form and an A as superscript represents the active form.

(R1) $$\text{x}+{\left(\text{C}{\text{a}}^{+2}\right)}_{4}.\text{CaM}\;\begin{array}{c} \overset{{\text{k}}_{10}}{\underset{}{\to }}\\ \overset{}{\underset{{\text{k}}_{1010}}{\leftarrow }} \end{array}\;\;{\text{x}}_{\text{P}}$$

(R2) $$\text{x}+{\text{x}}_{\text{P}}\;\;\begin{array}{c} \overset{{\text{k}}_{1}}{\underset{}{\to }}\\ \overset{}{\underset{{\text{k}}_{2}}{\leftarrow }} \end{array}\;{\text{C}}_{1}\;\overset{{\text{k}}_{3}}{\underset{}{\to }}\;\;2{\text{x}}_{\text{P}}$$

(R3) $${\text{X}}_{\text{P}}+\text{P}\;\;\overset{{\text{k}}_{4}}{\underset{}{\to }}\;\;\text{X}+\text{P}$$

(R4) $$\text{Y}+{\text{x}}_{\text{P}}\;\;\begin{array}{c} \overset{{\text{k}}_{5}}{\underset{}{\to }}\\ \overset{}{\underset{{\text{k}}_{55}}{\leftarrow }} \end{array}\;\;{\text{C}}_{2}\;\overset{{\text{k}}_{6}}{\underset{}{\to }}\;{\text{Y}}_{\text{P}}+{\text{X}}_{\text{P}}$$

(R5) $${\text{Y}}_{\text{P}}+\text{P}\;\;\overset{{\text{k}}_{7}}{\underset{}{\to }}\;\;\text{Y}+\text{P}$$

(R6) $${\text{Y}}_{\text{P}}+\text{T}\;\;\begin{array}{c} \overset{\text{k}8}{\underset{}{\to }}\\ \overset{}{\underset{{\text{k}}_{88}}{\leftarrow }} \end{array}{\text{C}}_{3}\;\;\overset{{\text{k}}_{9}}{\underset{}{\to }}\;\;{\text{Y}}_{\text{P}}+\text{X}+\text{T}$$

From above reactions following differential equations can be deduced.

(A-1) $$\begin{array}{c} \frac{\text{d}{\text{X}}_{\text{P}}}{\text{dt}}=-{\text{k}}_{1}*\text{X}*{\text{X}}_{\text{P}}+{\text{k}}_{2}*{\text{C}}_{1}+2*{\text{k}}_{3}*{\text{C}}_{1}-{\text{k}}_{4}*{\text{X}}_{\text{P}}*\text{P}+{\text{k}}_{55}*{\text{C}}_{2}+{\text{k}}_{6}*{\text{C}}_{2}\\ \;\;\;\;-{\text{k}}_{5}*\text{Y}*{\text{X}}_{\text{P}}+{\text{k}}_{10}*\text{x}*\text{U}-{\text{k}}_{1010}*{\text{X}}_{\text{P}}-\lambda_{1}*\left({\text{X}}_{\text{P}}-{\text{X}}_{\text{Pbasal}}\right) \end{array}$$

(A-2) $$\frac{\text{d}{\text{Y}}_{\text{P}}}{\text{dt}}=-{\text{k}}_{7}*{\text{Y}}_{\text{P}}*\text{P}+{\text{k}}_{6}*{\text{C}}_{2}-{\text{k}}_{8}*{\text{Y}}_{\text{P}}*\text{T}+{\text{k}}_{88}*{\text{C}}_{3}+{\text{k}}_{9}*{\text{C}}_{3}$$

(A-3) $$\begin{array}{c} \frac{\text{dX}}{\text{dt}}=-{\text{k}}_{1}*\text{X}*{\text{X}}_{\text{P}}+{\text{k}}_{2}*{\text{C}}_{1}+{\text{k}}_{4}*{\text{X}}_{\text{P}}*\text{P}+{\text{k}}_{9}*{\text{C}}_{3}+{\text{k}}_{10}*\text{x}*{\left(\text{C}{\text{a}}^{+2}\right)}_{4}.\text{CaM}-{\text{k}}_{1010}*{\text{X}}_{\text{P}}\\ \;\;\;\;-\lambda_{2}*\left(\text{X}-{\text{X}}_{\text{basal}}\right) \end{array}$$

(A-4) $$\frac{\text{d}{\text{C}}_{1}}{\text{dt}}={\text{k}}_{1}*\text{X}*{\text{X}}_{\text{P}}-{\text{k}}_{2}*{\text{C}}_{1}-{\text{k}}_{3}*{\text{C}}_{1}$$

(A-5) $$\frac{\text{d}{\text{C}}_{2}}{\text{dt}}={\text{k}}_{5}*\text{Y}*{\text{X}}_{\text{P}}-{\text{k}}_{55}*{\text{C}}_{2}-{\text{k}}_{6}*{\text{C}}_{2}$$

(A-6) $$\frac{\text{d}{\text{C}}_{3}}{\text{dt}}={\text{k}}_{8}*{\text{Y}}_{\text{P}}*\text{T}-{\text{k}}_{88}*{\text{C}}_{3}-{\text{k}}_{9}*{\text{C}}_{3}$$

### B. Model analysis and reduction

In the following equations, the dynamical variable X represents αCaMKII and Y represents the CPEB1. The P subscript represents the phosphorylated and active form. By using the pseudo-steady state assumptions the differential equations representing the complexes [C_1_-C_3_] can be eliminated:

(B-1) $${\text{C}}_{1}=\frac{{\text{k}}_{1}*\text{X}*{\text{X}}_{\text{P}}}{\left({\text{k}}_{2}+{\text{k}}_{3}\right)}$$

(B-2) $${\text{C}}_{2}=\frac{{\text{k}}_{5}*\text{Y}*{\text{X}}_{\text{P}}}{\left({\text{k}}_{55}+{\text{k}}_{6}\right)}$$

(B-3) $${\text{C}}_{3}=\frac{{\text{k}}_{8}*{\text{Y}}_{\text{P}}*\text{T}}{\left({\text{k}}_{88}+{\text{k}}_{9}\right)}$$

The unphosphorylated CPEB1 (Y) is related to phosphorylated CPEB1 (Y_P_).

(B-4) $$\text{Y}={\text{Y}}_{\text{T}}-{\text{Y}}_{\text{P}}$$

Where, Y_T _is the total amount of CPEB1. The αCaMKII molecules are either in free or in bound form, therefore, the total concentration of αCaMKII is given by following equation.

(B-5) $${\text{X}}_{\text{T}}=\text{X}+{\text{X}}_{\text{P}}+2*{\text{C}}_{1}+{\text{C}}_{2}$$

The differential equation representing the phosphorylated CPEB1 (Y_P_) from R_1_-R_6_

(B-6) $$\frac{\text{d}{\text{Y}}_{\text{P}}}{\text{dt}}=-{\text{k}}_{7}*{\text{Y}}_{\text{P}}*\text{P}+{\text{k}}_{6}*{\text{C}}_{2}-{\text{k}}_{8}*{\text{Y}}_{\text{P}}*\text{T}+{\text{k}}_{88}*{\text{C}}_{3}+{\text{k}}_{9}*{\text{C}}_{3}$$

By requiring a steady state $\frac{\text{d}{\text{Y}}_{\text{P}}}{\text{dt}}=0$ and substituting $\text{a}=\frac{\text{k}6*\text{k}5}{\left({\text{k}}_{55}+{\text{k}}_{6}\right)}$ we get

(B-7) $${\text{Y}}_{\text{P}}=\frac{\text{a}*{\text{Y}}_{\text{T}}*{\text{X}}_{\text{P}}}{\left({\text{k}}_{7}*\text{P}+\text{a}*{\text{X}}_{\text{P}}\right)}$$

Also from (B-1,B-2, B-3, B-4, B-5 and B-7) we can obtain the value of X.

(B-8) $$\text{X}=\frac{{\text{X}}_{\text{T}}-{\text{X}}_{\text{P}}-\text{N}*{\text{X}}_{\text{P}}*\left({\text{Y}}_{\text{T}}-\frac{\text{a}*{\text{X}}_{\text{P}}*{\text{Y}}_{\text{T}}}{{\text{k}}_{7}*\text{P}+\text{a}*{\text{X}}_{\text{P}}}\right)}{\left(1+2*\text{M}*{\text{X}}_{\text{P}}\right)}$$

Where, N and M are constants defined as $\text{N}=\frac{{\text{k}}_{5}}{\left({\text{k}}_{55}+{\text{k}}_{6}\right)},\text{M}=\frac{{\text{k}}_{1}}{\left({\text{k}}_{2}+{\text{k}}_{3}\right)}$

As described in the result section the equation 2 is as follows:

(2) $$\text{H}\left({\text{C}}_{3},{\text{X}}_{\text{T}}\right)={\text{k}}_{9}*{\text{C}}_{3}\;-\lambda *{\text{X}}_{\text{T}}$$

Substituting C3 from (B-3) in [2]

(B-9) $$\text{H}\left({\text{Y}}_{\text{P}},{\text{X}}_{\text{T}}\right)={\text{k}}_{9}*\left(\frac{{\text{k}}_{8}*\text{T}}{{\text{k}}_{88}+{\text{k}}_{9}}\right)*{\text{Y}}_{\text{P}}\;-\lambda *{\text{X}}_{\text{T}}$$

Placing Y_P _from (B-7) into (B-9)

(B-10) $$\text{H}\left({\text{X}}_{\text{P}},{\text{X}}_{\text{T}}\right)={\text{k}}_{9}*\left(\frac{{\text{k}}_{8}*\text{T}}{{\text{k}}_{88}+{\text{k}}_{9}}\right)*\left(\frac{\text{a}*{\text{Y}}_{\text{T}}*{\text{X}}_{\text{P}}}{\text{a}*{\text{X}}_{\text{P}}+{\text{P}}_{7}}\right)-\lambda *{\text{X}}_{\text{T}}$$

Where, P_7 _= P*k_7 _and P_4 _= P*k_4_. Defining, U = (Ca^+2^)_4_-CaM, $\text{b}=\left(\frac{{\text{k}}_{9}*{\text{k}}_{8}*\text{T}}{\left({\text{k}}_{88}+{\text{k}}_{9}\right)}\right)$ and c = b*Y_T _or $\text{c}={\text{Y}}_{\text{T}}*\left(\frac{{\text{k}}_{9}*{\text{k}}_{8}*\text{T}}{\left({\text{k}}_{88}+{\text{k}}_{9}\right)}\right)$

We obtain

(B-11) $$\text{H}\left({\text{X}}_{\text{P}},{\text{X}}_{\text{T}}\right)=\left(\frac{\text{c}*{\text{X}}_{\text{P}}}{{\text{X}}_{\text{P}}+{\text{P}}_{7}/\text{a}}\right)-\lambda *{\text{X}}_{\text{T}}$$

The equation 1 describes the rate of change of total concentration of αCaMKII (X_T_) is obtained from the balance between the new synthesis of αCaMKII and its degradation. This equation is further transformed to the approximate solution as shown by equation 4. Similarly, from R_1_-R_6 _an equation representing the dynamics of phosphrylated αCaMKII can be constructed.

(B-12) $$\begin{array}{c} \frac{\text{d}{\text{X}}_{\text{P}}}{\text{dt}}=-{\text{k}}_{1}*\text{X}*{\text{X}}_{\text{P}}+{\text{k}}_{2}*{\text{C}}_{1}+2*{\text{k}}_{3}*{\text{C}}_{1}-{\text{k}}_{\text{4}}*{\text{X}}_{\text{P}}*\text{P}+{\text{k}}_{55}*{\text{C}}_{2}+{\text{k}}_{6}*{\text{C}}_{2}\\ \;\;\;\;-{\text{k}}_{5}*\text{Y}*{\text{X}}_{\text{P}}+{\text{k}}_{10}*\text{X}*\text{U}-{\text{k}}_{1010}*{\text{X}}_{\text{P}} \end{array}$$

The equation B-12 is further simplified in only two variables i.e., X_T _and X_P _by placing the $\frac{\text{d}{\text{Y}}_{\text{P}}}{\text{dt}}=0$ and putting the values of X, C_1_, C_2_, Y, and Y_P _from B-1 to B-8 and further simplifying we get the following expression.

(B-13) $${\text{X}}_{\text{T}}={\text{I}}_{1}+{\text{I}}_{2}+{\text{I}}_{3}+{\text{I}}_{4}$$

The I_1_,I_2_,I_3 _and I_4 _are defined as follows:

(B-14) $${\text{I}}_{1}={\text{D}}_{\text{I}}.*{\text{X}}_{\text{P}}./\left({\text{a}}_{1}.*{\text{X}}_{\text{P}}+{\text{a}}_{2}\right)$$

(B-15) $${\text{I}}_{2}=\left[{\text{D}}_{\text{I}}.*{\text{X}}_{\text{P}}.^{2}./\left({\text{k}}_{3}.*{\text{X}}_{\text{P}}./2+{\text{k}}_{3}.*{\text{a}}_{2}./\left(2*{\text{a}}_{1}\right)\right)\right]$$

(B-16) $${\text{I}}_{3}={\text{X}}_{\text{P}}$$

(B-17) $${\text{I}}_{4}={\text{X}}_{\text{P}}.*{\text{Y}}_{\text{T}}./\left({\text{k}}_{6}./{\text{d}}_{1}+{\text{X}}_{\text{P}}./{\text{k}}_{7}\right)$$

The expression in B-14 to B-17 are defined as k_7 _= k_7 _*P, k_4 _= k_4 _*P,a_1 _= (k_3 _*k_1 _)./(k_2 _+ k_3 _) a_2 _= k_10 _*U, D_I _= k_4 _+ k_1010 _, d_1 _= (k_6 _.*k_5_)./(k_55 _+ k_6_)

The expression of B-13 gives us a function where X_T _= f(X_P_), It is almost impossible to invert this function to get X_P _= f^-1^(X_T_)so we numerically approximated it through function fitting.

Equation B-12 with the aid of equation B1-B 13 can be transformed to following polynomial in terms of X_P_.

(B-18) $${\left({\text{X}}_{\text{P}}\right)}^{4}+{\text{z}}_{12}*{\left({\text{X}}_{\text{P}}\right)}^{3}+{\text{z}}_{13}*{\left({\text{X}}_{\text{P}}\right)}^{2}+{\text{z}}_{14}*{\text{X}}_{\text{P}}=0$$

(B-19) $${\text{z}}_{1}=\text{a}*\text{c}*\text{M}*{\text{k}}_{3}*{\text{P}}_{7}$$

(B-20) $${\text{z}}_{2}=\text{a}*\text{c}*{\text{k}}_{10}*\text{U}*{\text{P}}_{7}$$

(B-21) $${\text{z}}_{3}={\text{a}}^{2}*\text{c}*\text{M}*{\text{k}}_{3}$$

(B-22) $${\text{z}}_{4}={\text{a}}^{2}*\text{c}*\text{F}*{\text{k}}_{10}*\text{U}$$

(B-23) $${\text{z}}_{5}=2*{\text{P}}_{4}*\text{M}*\text{F}+2*\text{M}*{\text{k}}_{1010}*\text{F}+\text{F}*\text{M}*{\text{k}}_{3}$$

(B-24) $${\text{z}}_{6}=2*{\text{P}}_{4}*{\text{P}}_{7}*\text{M}+2*\text{M}*{\text{P}}_{7}{*}_{\text{k}1010}+\text{F}*{\text{P}}_{4}+\text{F}*{\text{k}}_{1010}+\text{M}*{\text{k}}_{3}*{\text{P}}_{7}+\text{F}*{\text{k}}_{10}*\text{U}+\text{N}*\text{M}*{\text{k}}_{3}*{\text{Y}}_{\text{T}}$$

(B-25) $${\text{z}}_{7}={\text{P}}_{7}*{\text{P}}_{4}+{\text{P}}_{7}*{\text{k}}_{1010}+{\text{k}}_{10}*\text{U}*{\text{P}}_{7}+\text{N}*{\text{k}}_{10}*\text{U}*{\text{P}}_{7}*{\text{Y}}_{\text{T}}$$

(B-26) $${\text{z}}_{8}=\text{a}*{\text{z}}_{5}$$

(B-27) $${\text{z}}_{9}=\text{a}*{\text{z}}_{6}+{\text{P}}_{7}*{\text{z}}_{5}-{\text{z}}_{3}$$

(B-28) $${\text{z}}_{10}=\text{a}*{\text{z}}_{7}+{\text{P}}_{7}*{\text{z}}_{6}-{\text{z}}_{1}-{\text{z}}_{4}$$

(B-29) $${\text{z}}_{11}={\text{p}}_{7}*{\text{z}}_{7}-{\text{z}}_{2}$$

(B-30) $${\text{z}}_{12}=\frac{{\text{z}}_{9}}{{\text{z}}_{8}}$$

(B-31) $${\text{z}}_{13}=\frac{{\text{z}}_{10}}{{\text{z}}_{8}}$$

(B-32) $${\text{z}}_{14}=\frac{{\text{z}}_{11}}{{\text{z}}_{8}}$$

Where, the coefficients *z*_12_*,z*_13 _and *z*_14 _are defined by following expressions (B-19-B-32). The equation (B-18) is obtained through further simplifying (B-13) such that a fourth order polynomial is obtained in terms of X_P_. This 4'th order polynomial has an analytical solution because it has no zero order term and therefore has one solution X_P _= 0, and the other solutions are the solutions of a third order polynomial.

## Results

### Analysis of the model of αCaMKII synthesis through polyadenylation

**Figure 1:**
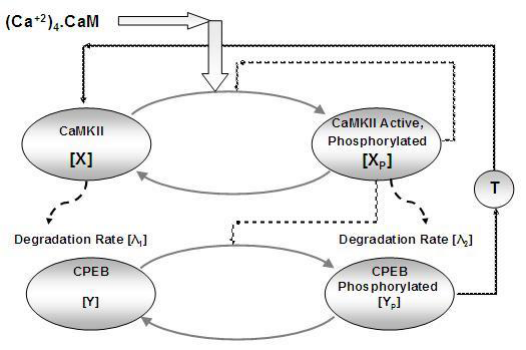
**The simplified model of CPEB1 mediated polyadenylation of αCaMKII through a self-sustaining αCaMKII-CPEB 1 molecular loop**. The αCaMKII, molecule can be inactive, or active and phosphorylated state, while CPEB1 can be in unphosphorylated and phosphorylated states. The αCaMKII molecule in both these states degrades with a certain degradation rate. The active and phosphorylated CaMKII phosphorylate the CPEB1 molecule, which through polyadenylation of αCaMKII mRNA generates the new CaMKII molecule. Here, the synthesis of new αCaMKII molecule is represented by a translation step (T) which, describe the multi-step translation process through a single biochemical reaction R6 as described in appendix.

Our simplified model of a self-sustained polyadenylation of αCaMKII-mRNA (Figure 1) is based on biochemical interactions between a plasticity related kinase αCaMKII and its translational regulator CPEB1 through a positive feedback. This model of polyadenylation based translation of αCaMKII-mRNA (Figure 1) is composed of two molecular components which interact through a closed loop. 1) The αCaMKII protein which can be in two states: inactive, and phosphorylated, active. 2) The CPEB1 a translational regulator to regulate the polyadenylation, can be either in the phosphorylated or unphosphorylated state. The phosphorylated CPEB1 promotes the translation at pre-initiation phase through polyadenylation. Here, in this simple model we assume that CPEB1 is phosphorylated only by active and phosphorylated αCaMKII. In this molecular scheme the αCaMKII protein is removed at a certain degradation rate [27]. The synthesis of new αCaMKII protein regulated by polyadenylation provides the necessary compensation for the amount removed due to degradation. Our model shows how the concentration of αCaMKII can be maintained at multiple levels despite the synthesis of new molecules and removal due to protein degradation.

This system is described by a set of differential equation (**Method A**). We use notation in which CaMKII is denoted as X, and phosphorylated αCaMKII as X_P_.

Similarly CPEB is denoted as Y and phosphorylated CPEB1 as Y_P_. On purpose we have chosen a simplified version of these components, when compared to our previous work [28] in order to gain intuition into the behavior of the system while keeping the bistability. We can easily show that for appropriate parameters this system is bistable. However, gaining an intuitive understanding of this bistability is difficult even in this simplified system, because the dimensionality is still too large.

The two-state assumption of the αCaMKII is a significant simplification compared to more realistic multi-state models and was chosen to enable simpler analysis. The synthesis of new proteins in our proposed molecular model is controlled by the phosphorylated CPEB1 molecules. The translation model is also a simplified representation of a complex molecular system. The aim of this paper is to generate a much reduced dynamical model that can nevertheless capture the qualitative behavior of the system, and provide us with a more intuitive understanding of dynamical behavior of the system.

One level of simplification is to set the derivatives of the three complexes (C_1_-C_3_) to zero (**Method B**), to obtain a pseudo steady state approximation, similar to that used in the standard michaelis-menten approximation. This reduces our system to 3 dynamical equations, and three functional expressions, as described by equations 1, B-6, and B-12. This approximation simplifies the dynamics, and can be used to more easily find the steady states of this system numerically. We can further approximate this system by a single differential equation. The key simplifying assumption required here is the following equation:

(1) $$\frac{\text{d}{\text{X}}_{\text{T}}}{\text{dt}}={\text{k}}_{9}*{\text{C}}_{3}-\lambda *{\text{X}}_{\text{T}}$$

Where, C_3_, as described by equation B-3 in the method B, is the concentration of the phosphorylated form of CPEB1, bound to the translation machinery, or in other words the concentration of active translation machinery available for producing new αCaMKII. Here k_9 _is the forward rate of generating new αCaMKII. This equation assumes that αCaMKII degrades at the same rates both in free and bound forms. The X_T _in above equation (Eq. 1) is the concentration of total αCaMKII. In our detailed system of equations (Method A) we do not assume the degradation of complexes. However, this approximation holds if the relative concentration of complexes at steady state is small.

Defining $\frac{\text{d}{\text{X}}_{\text{T}}}{\text{dt}}=\text{H}\left({\text{C}}_{3},X_{T}\right)$ we have that:

(2a) $$\text{H}\left({\text{C}}_{3},{\text{X}}_{\text{T}}\right)={\text{k}}_{9}*{\text{C}}_{3}-\lambda *{\text{X}}_{\text{T}}$$

By using a pseudo steady state approximation on some of the faster dynamical variables, we obtain (Method B) that:

(3) $$\text{H}\left({\text{X}}_{\text{P}},{\text{X}}_{\text{T}}\right)=\left(\frac{\text{c}*{\text{X}}_{\text{P}}}{{\text{X}}_{\text{P}}+{\text{P}}_{7}/\text{a}}\right)-\lambda *{\text{X}}_{\text{T}}$$

Where the parameters 'c', 'P7' and 'a', are defined in Method B. The parameter c is linearly dependent on Y_T_, and is inversely proportional to degradation rate *λ*. It shows that amount of total αCaMKII increases with increase in translation (either due to increase in translation rate or concentration of translation machinery "T") and decreases with increase in degradation rate. The K_m1/2 _of this process is *P*_7_/a and depends on phosphatses and dephosphorylation rate of CPEB1.

We call the first function of RHS of equation 3 the synthesis function and the second one a degradation function. We can rewrite equation 3 as

(4) $$\text{H}\left({\text{X}}_{\text{P}},{\text{X}}_{\text{T}}\right)+\text{G}\left({\text{X}}_{\text{P}}\right)-\text{F}\left({\text{X}}_{\text{T}}\right)$$

Where, G is a synthesis function, and F a degradation function, and the dynamics are then understood as a balance between synthesis and degradation. If we can derive a function that relates X_p _to X_T_, i.e., X_P _= g (X_T_), then equation 4 could be converted to equation of a single dynamical variable:

(5) $$\text{H}\left({\text{X}}_{\text{T}}\right)=\text{G}\left(\text{g}\left({\text{X}}_{\text{T}}\right)\right)-\text{F}\left({\text{X}}_{\text{T}}\right)=\text{G'}\left({\text{X}}_{\text{T}}\right)-\text{F}\left({\text{X}}_{\text{T}}\right)$$

and we would have reduced the 6 coupled ODE's to a single ODE:

(6) $$\frac{\text{d}{\text{X}}_{\text{T}}}{\text{dt}}=\text{G'}\left({\text{X}}_{\text{T}}\right)-\text{F}\left({\text{X}}_{\text{T}}\right)$$

Although we have an exact expression for X_T _as a function of X_P _[(Method B equation 13)], it is difficult to analytically invert it to obtain an analytical expression for X_P _= g ( X_T_). Instead it is easy to invert this numerically using a higher order polynomial fit.

Using this fit.

(7) $${\text{X}}_{\text{P}}=\overset{9}{\underset{\text{i}=1}{\sum }}{\text{a}}_{\text{i}}*{{\text{X}}^{\text{i}}}_{\text{T}}=\text{h}\left({\text{X}}_{\text{T}}\right)$$

The fitting performance of this equation is shown in additional file 1, Figure S_1_.The solid black line in additional file 1, Figure S_1 _represents the original function {Method B B-13}, while the dotted red line represents the fit from equation 7. Thus we reduce the 6 differential equations to a single approximate ODE of the form described in equation 6, where:

(8) $$\text{G'}\left({\text{X}}_{\text{T}}\right)=\left(\frac{\text{c}*\text{h}\left({\text{X}}_{\text{T}}\right)}{\text{h}\left({\text{X}}_{\text{T}}\right)+{\text{P}}_{7}/\text{a}}\right)$$

Here, the X_P _is obtained from fitting function 7 and F (X_T_) is given by following equation.

(9) $$\text{F}\left({\text{X}}_{\text{T}}\right)=\lambda *{\text{X}}_{\text{T}}$$

**Figure 2:**
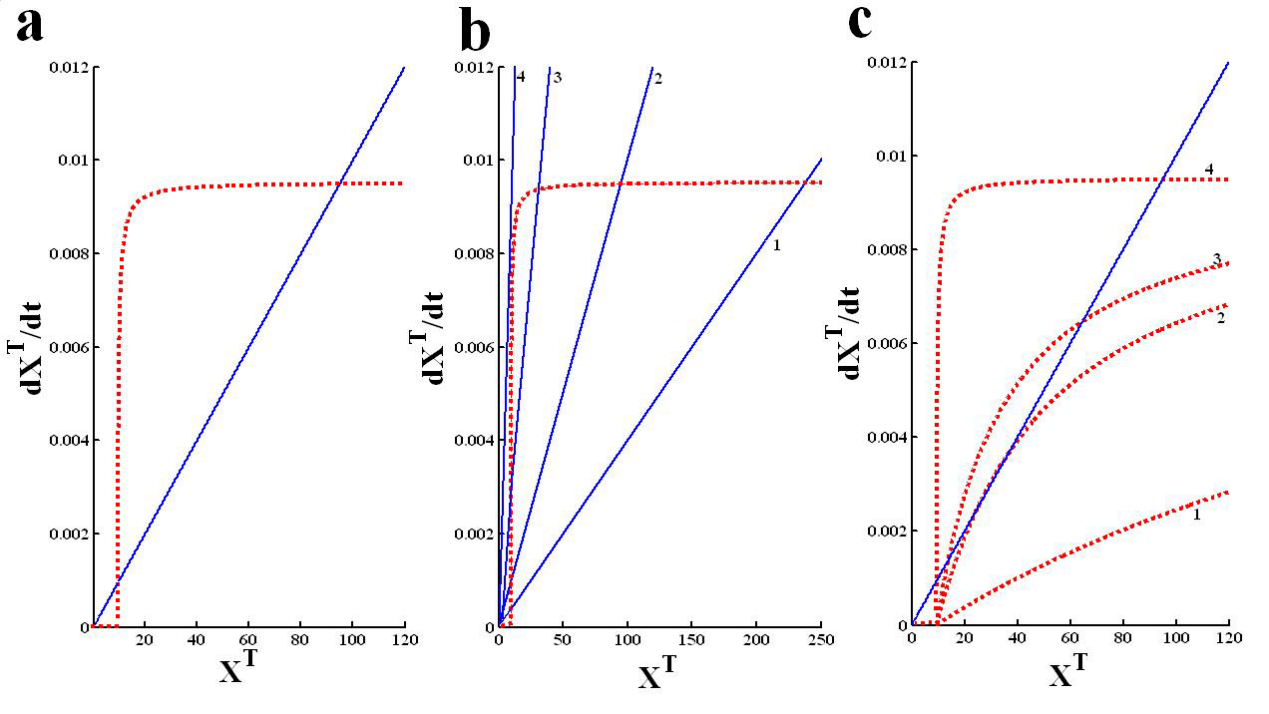
**The approximate analytical solution of αCaMKII-CPEB1 molecular loop**. It is obtained through graphically locating all the steady state solutions of single differential equation 1. This equation describes the rate of change of total concentration of αCaMKII (X_T_) and its functional relation between the new synthesis and degradation of CaMKII. The steady state form of equation 1 is transformed into two functions F (X_T_) and G (X_T_). The function F (X_T_) (**solid blue curve**) basically represents the net αCaMKII degradation, whereas the modified function G'(X_T_) = G [g (X_T_)] (**dotted red curve**) represent the net generation of αCaMKII. **(a) **This analytical solution is developed by setting all parameters as described in table 1. Graphs of both functions intersect at three locations which are characterized as steady state solutions of equation 1 and provide the approximate analytical solution. The upper steady state solution is located at X_T _= 95, while the unstable solution is located at X_T _= 9.4, and lower steady state solution at X_T _= 0.0001. **(b) **The effect of degradation rate on analytical solution. Four different degradation rates are selected *λ *= 0.00006 s- (**solid blue line #1, bistable system**), λ, = 0.0001 s^-1 ^(**solid blue line #2 bistable system**), *λ *= 0.0003 s^-1^(**solid blue line #3 bistable system**) and *λ *= 0.0006 s^-1 ^(**solid blue line #4 mono-stable system**). **(c) **The effect of CPEB1 activation parameter k_5 _on analytical solution. Four different values of CPEB1 activation are selected k_5 _= 0.00001*μ*M^-1^.s^-1 ^(**dotted red line #1, mono-stable system**), k_5 _= 0.00006*μ*M^-1^.s^-1 ^(**dotted red line #2 bistable system**), k_5 _= 0.0001*μ*M^-1^.s^-1 ^(**dotted red line #3 bistable system**) and *λ *= 0.0072 *μ*M^-1^s^-1 ^(**dotted red line #4 bistable system**).

In order, to gain some intuitive understanding of this system we graphically plot the two terms in equation 6 (Figure 2a). The function G is shown as dotted red line and F as solid blue line. This graphical representation (Figure 2a) provides a very simple intuitive explanation of the behavior of the system. The function G' can be seen as the synthesis function (a source) and the function F as the degradation function (a sink). The intersections between these two curves are the systems fixed points. For low values of X_T _there is hardly any synthesis, and degradation dominates, so the system converges to the low fixed point at zero. At higher values of X_T _there is an abrupt rise in the level of protein synthesis, which quickly saturates. The cross between G and F in the quickly rising portion of G is the unstable fixed point, below it degradation dominates and above it synthesis dominates, and the level of X_T _increases until it reaches the third intersection, which is the upper stable fixed point. Due to the saturation of G, at higher levels of X_T_, degradation dominates again, resulting in a convergence from above to the upper stable fixed point. Changing the degradation rate will simply change the slope of the F function, and changing synthesis and activation parameters, will quantitatively change the shape of the G' function.

Next we show the impact of changing some of the system parameters on the steady state solution characteristics of equation 6 (Figure 2b and 2c). Changing the degradation rate only affects the degradation curve (Figure 2b**solid blue line**). As the degradation rate is increased from slower (Figure 2b**solid blue line 1**) to much faster (Figure 2b**solid blue line 4**) the system moves from robustly bistable to a mono-stable system. Thus, at much faster degradation the amount of CaMKII generated is not enough to compensate for the loss due to large degradation rate therefore, system has only lower stable steady state solution (Figure 2b**solid blue line 4**) as the CaMKII degradation rate is decreased a balance between new synthesis and degradation is restored and system becomes a bistable switch (Figure 2b**solid blue lines 2-4)**. As the degradation rate is decreased, and the system as shifts from mono-stable to bistable, the lower fixed point does not change, the value of X_T _at the unstable fixed point is increased, and the value of X_T _at the upper fixed point first rapidly increases and then plateaus (Figure 2b**solid blue line 2,3,4**).

Other parameters affect only the synthesis curve (G'). One such example is parameter k_5_, which quantifies the CaMKII mediated activation of CPEB1 (Figure 2c). When this activation parameter is set at low value (Figure 2c**dotted red line 4**) the system has only lower stable steady state solution. However, as the activation rate of CPEB1 is increased, more active CPEB1 is available, which in turn stimulates new protein synthesis thus shifting the synthesis curve in upward direction (Figure 2c**dotted red line 3,2,1**) and shifting the system from mono-stable to robustly bistable state.

If we are only interested in the steady states of this system we can set all the derivatives to zero and obtain the following forth order polynomial to describe the steady states of the system:

(10) $${\left({\text{X}}_{\text{P}}\right)}^{4}+{\text{z}}_{12}*{\left({\text{X}}_{\text{P}}\right)}^{3}+{\text{z}}_{13}*{\left({\text{X}}_{\text{P}}\right)}^{2}+{\text{z}}_{14}*{\text{X}}_{\text{P}}=0$$

Where, the coefficients z_12_-z_14 _are defined in method B. The derivation leading to this result is explained in method B. This equation is more precise than equation 6 because here we avoided the need to use the polynomial fit for the inversion. However, this equation can only account for the fixed points, and is unable to account for system dynamics. This 4'th order polynomial shows that this system has 4 steady states, one is always at zero because there is no zero order term. The signs of the coefficients determine how many real solutions this polynomial could have [29] and biologically we are only interested in positive real solutions.

### Comparison of the different approximations to the full model

Our aim here is to describe the behavior of the synthesis-degradation loop (Figure 1) and the various approximations used to simplify it. We can integrate the dynamics either using the full system of ODE's (Method A, A1-A6) or by using the reduced three dimensional systems (Method B, equations 6, B-6, B12), or the approximate one dimensional system (equation 6). The fixed points, and their parameter dependence can be found either by numerical bifurcation analysis of the full system (Method A, A1-A6), the reduced three dimensional system (6, B-6, B12), the approximate 1D system (equation 6) or by the 4'th order polynomial [10]. The first three methods can be used for obtaining the system dynamics and the last method only for the fixed points.

**Figure 3:**
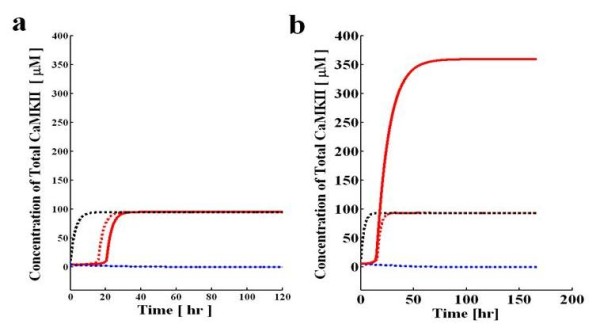
**The dynamic characteristics of aCaMKII-CPEB 1 molecular loop**. These characteristics are developed through three alternative models. First model is based on differential equations A1-A6 (**Full model, represented by solid red line (up state) and solid blue line (down state**)). Second model is based on differential equation 1, B-6 and B-9 (**Reduced model, represented by dotted red line (up state) and dotted blue line (down state**)). Third model is based only on a single differential equation (equation 6) (**Single differential equation model, represented by dotted black line (up state) and dotted blue line (down state**)). System moves from down to up-regulated state through a 10 second application of (Ca^+2 ^)_4_-CaM pulse. The dynamic simulations are carried out with two different set of parameters. For one set of parameters the dynamic from three alternative models converge to same upper and lower steady states **(a)**. In this case the dynamics converge to same upper steady state at X_T _= 95 and lower steady state at X_T _= 0.0 For second selection of parameters the dynamic results from three alternative models does not converge to same upper steady state **(b) **although the lower steady state remains the same for all three models. Here, the dynamics from full model converge to upper steady state at X_T _= 360 and lower state at X_T _= 0.0001 (Figure 3 b, **solid red line (up state) and solid blue line (down state**)), whereas the dynamics from reduced model (**dotted red line (up state) and dotted blue line (down state**)) and single differential equation model (**dotted black line (up state) and dotted blue line (down state**)) converge to upper steady state at X_T _= 95 and lower state at X_T _= 0.0001.

In order, to further compare the three dynamical equation methods we first analyzed the dynamical properties of this system and then the steady state solution characteristics of this loop with respect to certain system parameters. The dynamics of this system (Figure 3) is analyzed with two different set of parameters. The first set of parameters (Condition I) represent a case where the approximation of equation 1 is a good aproximation (Figure 3a), whereas the second set of parameters (Condition II) represents a case where this approximation does not hold (Figure 3b). For condition I the approximation holds since the amount of αCaMKII bound in biochemical complexes is negligible, whereas for condition II a significant portion is trapped in biochemical complexes. We obtain these different conditions by setting the different values of auto-phosphorylation parameter k_3_. For condition I we set k_3 _= 500*μ*M^-1 ^s^-1^, whereas for condition II we set this parameter at 0.5 *μ*M^-1 ^s^-1^. The values of all the other parameters are identical in both conditions (Table 1). For all these cases a 10 second (Ca^+2 ^)_4_-CaM pulse is used to provide the necessary stimulus to drive system from the basal to the up-state.

**Table 1:** The numerical values of different parameters of bistable molecular loop.

Parameter	Description	Value/Range
***λ***_**1**_	CaMKII degradation rate (In-active state)	[0.0001 (sec-^1^)] 0.001-0.00002(sec-^1^)
*λ*_2_	CaMKII degradation rate (Active, phosphorylated)	[0.0001 (sec-^1^)] 0.001-0.00002(sec-^1^)
**k**_**1**_	Association rate constant (Ca^+2^)_4_-CaM/CaMKII binding	0.0011 (μM- ^1^sec-^1^)
**k2**	Dissociation rate constant CaCaM.CaMKII	14 (sec-^1^)
**k**_**1**_	Association rate constant CaMKII^A^_P_/CaMKII^A^_P _binding	0.085; (μM^-1 ^sec- )
**k**_**2**_	Dissociation rate constant CaMKII^A^_P_.CaMKII^A^_P_	0.143 (sec^-1^)
**k**_**3**_	Rate constant for Phosphorylation	500 (sec-^1^)
**k**_**4**_	Rate constant for CaMKII^A^_P _dephosphorylation	0.0012 (sec-^1^)
**k**_**5**_	Association rate constant CaMKII^A^_P_/CPEB1 binding	0.0072;(μM-^1^sec-^1^)
**k55**	Dissociation rate constant CaMKII^A^_P _.CPEB1	20(sec-^1^)
**k**_**6**_	Rate constant for CPEB1 Phosphorylation	0.962(sec-^1^)
**k10**	Association rate constant (Ca^+2^)_4_-CaM/CaMKII binding	0.001 (μM ^1^sec-^1^)
**k1010**	Dissociation rate constant CaCaM.CaMKII	0.8 (sec-^1^)
**k**_**7**_	Rate constant for CPEB1 dephosphorylation	0.012(sec-^1^)
**k**_**8**_	Association rate constant PCPEB/E binding	0.08(μM- ^1^sec-^1^)
**k88**	Dissociation rate constant PCPEB1.E	10(sec-^1^)
**k**_**9**_	Synthesis Rate of New CaMKII molecule through polyadenylation	0.08(sec-^1^)
**Xbasal**	Basal concentration of CaMKII	0.0001 μM

For condition I the dynamics from all three alternative models converge to same upper and lower steady states (X_T _= 95 for upper steady state and X_T _= 0.0001 for lower steady state Figure 3a. The full model is represented by solid red line (up state) and solid blue line (down state). The various dashed lines present the lower dimensional approximations. We see that even though in condition I the steady state behavior is well approximated by the reduced systems, the dynamics are not. By setting derivatives to zero and replacing some dynamical variables by functions we have produced reduced dynamics that are faster than the full model dynamics. These types of approximations can produce reasonable dynamics if there is a clear separation of time scales, in which case setting the derivatives of the fast dynamics to zero causes only minor differences in the dynamics. Here, although degradation is the slowest dynamical variable, other processes such as dephosphorylation can also be slow, and therefore there is no clear time scale separation. For condition II (Figure 3b) even the steady state behavior of the systems is not well approximated by the reduced equations.

**Figure 4:**
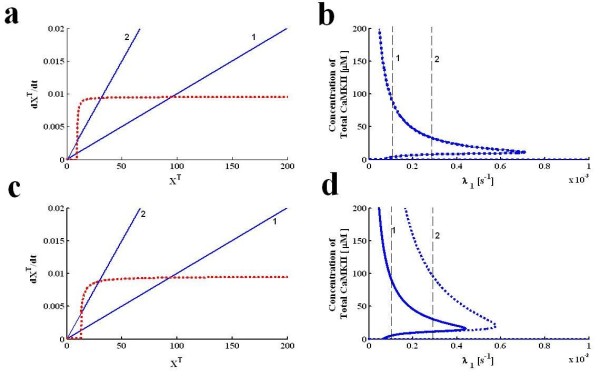
**Steady state solution characteristics of molecular loop with respect to parameter *λ***. This contain an analytical solution **(a, c) **and numerical/analytical bifurcation diagrams **(b, d)**. Approximate analytical solution is developed through graphing equation 6, whereas the numerical/analytical bifurcation diagrams are developed through tracking the steady state behavior of all three models with respect to the degradation rate. Two different set of parameters are compared. For first set of parameters the approximate solution and numerical/analytical bifurcation diagrams from three alternative models converge to same upper, stable steady state solution branch **(a, b)**. For the second set of parameters the approximate solution and numerical/analytical bifurcation diagrams from three alternative models does not converge to same upper, stable steady state and solution branch**(c, d)**. The approximate analytical solution for first set of parameters **(a) **is located at two different degradation rates **(The solid blue line#"1" represent ***λ *= 0.0001s^-1 ^**and other solid blue line#"2" represent ***λ *= 0.0003 s^-1 ^**)**. This method locates the upper stable steady state at X_T _= 95, while the lower and unstable steady state are at X_T _= 0 and X_T _= 9.4 when degradation rate is set at 0.0001 s^-1 ^(**curve 1**). As degradation rate is increased to 0.0003 s^-1 ^(**curve 2**) the new solution is located at X_T _= 31 (upper stable steady state), X_T _= 0.0001 (lower stable steady state) and X_T _= 9.2 (unstable steady state). The numerical and analytical bifurcation diagrams for first set of parameters (**b**) are developed through tracking the steady state behavior of all three models. Three bifurcation diagrams exactly match with each other for the entire range of degradation parameter (**dotted blue line represent the bifurcation diagram based on full model, whereas solid blue lines represent the bifurcation diagrams based on reduced three differential equation and a single equation model**). The approximate analytical solution for second set of parameters **(c) **is also located at two different degradation rates **(The solid blue curve "1" represent ***λ *= 0.0001s^-1^**, and other solid blue curve "2" represent ***λ *= 0.0003 s^-1^**)**. This method locates the upper stable steady state at X_T _= 95, while the lower and unstable steady state are at X_T _= 0.0001 and X_T _= 9.4 when degradation rate is set at 0.0001s-^1 ^(**curve 1**). As degradation rate is increased to 0.0003 s^-1^(**curve 2**) the new solution is located at X_T _= 31 (upper stable steady state), X_T _= 0.0001 (lower stable steady state) and X_T _= 9.2 (unstable steady state). The numerical and analytical bifurcation diagrams for second set of parameters (**d**) are developed. The bifurcation diagram from full model does not match with the bifurcation diagrams from reduced and single equation model.

Next, we analyze the dependence of the steady state solution of this loop on different system parameters. This is similar to what we do in Figure 2, but here we show the full bifurcation diagrams resulting from a change in parameters. We also compare cases where the approximations used are appropriate or not. First, we select the degradation rate (*λ*) of αCaMKII as a bifurcation parameter. Here, degradation represents the removal of αCaMKII either by general cellular degradation pathway or by diffusion from a certain specific location e.g., active synapses. We analyzed the effect of degradation parameter on the characteristics of polyadenylation loop under the two different set of parameters (conditions I and II). We show results of this analysis in Figure 4. On the left we show the G' and F functions (Figure 4a,c), and on the right the complete bifurcation diagrams in terms of *λ *(Figure 4b,d). Figures 4 a, b are for condition I and 4 c, d is for condition II.

We show the F functions for two different values of *λ*, where "1" denotes λ = 0.0001s^-1 ^and "2" denotes *λ *= 0.0003s^-1^. By this method we can graphically locate the values of X^T ^at the fixed points. For condition I the upper stable steady state at X_T _= 95, while the lower and unstable steady state are at X_T _= 0.0001 and X_T _= 9.4 when degradation rate is set at 0.0001 s^-1 ^(**curve "1"**, Figure 4a). As the degradation rate is increased to 0.0003 s^-1 ^(**curve "2"**, Figure 4a) the new solution is located at X_T _= 31 (upper stable steady state), X_T _= 0 (lower stable steady state) and X_T _= 9.2 (unstable steady state). Numerical bifurcation diagrams (Figure 4b) are developed through tracking the steady state behavior of all three levels of simplification with respect to the degradation rate of αCaMKII. The solutions contained in these bifurcation diagrams are tracked through a numerical bifurcation package [30], for the 6 and three dimensional models, by simply finding the cross-over in the one dimensional model, and by finding zeros of a polynomial in the polynomial approximation. The four bifurcation diagrams are nearly identical for the entire range of degradation parameter. In contrast, to these results when simulations are carried out with second set of parameters (condition II) the steady state solution characteristics of full scale model (Figure 4d**dotted blue line represent the bifurcation diagram based on full model) **does not match either with the three state reduced model or single equation model or the polynomial model (Figure 4d**solid blue lines represent the bifurcation diagrams based on reduced three differential equation and a single equation model**). What these results also indicate is that the most significant approximation made here is in equation 1, and it fails when the conditions of this approximation are not met.

**Figure 5:**
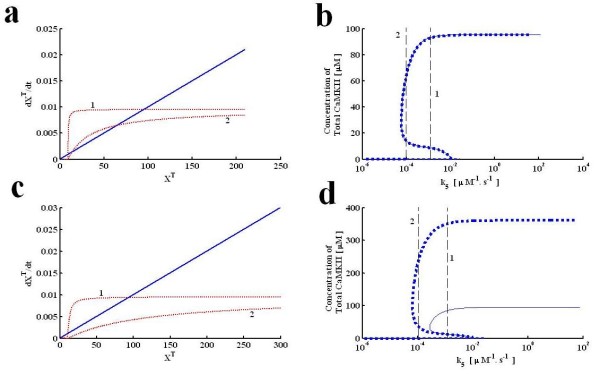
**Steady state solution characteristics of molecular loop with respect to CPEB1 activation parameter k**_**5**_. The parametric steady state solution characteristics are developed through an approximate analytical solution **(a, c) **and numerical/analytical bifurcation diagrams **(b, d)**. The approximate analytical solution is developed through graphing equation 6, whereas the numerical/analytical bifurcation diagrams are developed through tracking the steady state behavior of all three models with respect to k_5_. Two different set of parameters are compared. For first set of parameters the approximate solution and numerical/analytical bifurcation diagrams from three alternative models converge to same upper, stable steady state solution branch **(a, b)**. For the second set of parameters the approximate solution and numerical/analytical bifurcation diagrams from three alternative models does not converge to same upper, stable steady state and solution branch **(c, d)**. The approximate analytical solution for first set of parameters **(a) **is located at two different values of rate constant *k5 ***(The dotted red curve "1" represent k5 **= 0.0072 *μ*M^-1^*.s*^-1^**, second dotted red curve "2" represent k5 **= 0.0001 *μ*M^-1 ^.s^-1 ^). This method locates the upper stable steady state at X_T _= 95, while the lower and unstable steady state are at X_T _= 0.0001 and X_T _= 9.4 when k_5 _is set at 0.0072*μ*M^-1 ^.s^-1 ^(**curve "1"**). As k_5 _is decreased to 0.0001 new solution is located at X_T _= 70 (upper stable steady state), X_T _= 0.0001 (lower stable steady state) and X_T _= 16 (unstable steady state). The numerical and analytical bifurcation diagrams for first set of parameters (**b**) are developed through tracking the steady state behavior of all three models with respect to k5. The three bifurcation diagrams are exactly matching with each other for the entire range of activation parameter (**dotted blue line represent the bifurcation diagram based on full model, whereas solid blue lines represent the bifurcation diagrams based on reduced three differential equation and a single equation model**). The approximate analytical solution for second set of parameters **(c) **is also located at two different values of rate constant k_5 _**(The dotted red curve "1" represent k**_**5 **_= 0.0072 *μ*M^-1^*.s*^-1^**, second dotted red curve "2" represent k5 **= 0.0001 *μ*M^-1^.s^-1^**)**. The numerical and analytical bifurcation diagrams for second set of parameters (**d**) are developed through tracking the steady state behavior of all three models with respect to k5. Full model bifurcation diagram does not match with reduced and single equation model.

We also analyzed the steady state solution characteristics of this molecular switch with respect to CPEB1 activation parameter k_5_. First, the simulations are implemented with parameters from condition I (Figure 5, a, b) and then with condition II (Figure 5, c, d). Here, again the steady state solution characteristics of the full scale model do not match (Figure 5d**dotted blue line represent the bifurcation diagram based on full model, whereas solid blue lines represent the bifurcation diagrams based on reduced three differential equation and a single equation model**) with reduced versions when condition two is implemented, however, with parameters representing the condition one there is a complete match (Figure 5b**dotted blue line represent the bifurcation diagram based on full model, whereas solid blue lines represent the bifurcation diagrams based on reduced three differential equation and a single equation model**).

## Discussion

We have postulated that a translation-activation loop can form a bistable switch that could explain the mechanism for maintaining long term memories. Here, we explore a specific case of this hypothesis composed of a positive feedback loop between αCaMKII and its translation regulator CPEB1. The possibility of a positive feedback loop is confirmed by two previous experimental observations (a) phosphorylation of CPEB1 regulates the synthesis of αCaMKII molecules through polyadenylation of αCaMKII-mRNA [20] (b) αCaMKII phosphorylates the CPEB1 molecule [25,26]. Thus, both the αCaMKII and CPEB1 interact through a closed loop. Based on the elementary enzyme kinetics (Method A) the dynamics of this loop can be characterized through a high dimensional system of ordinary differential equations. One can study the characteristics of such a system by numerically integrating these differential equations and with different initial conditions and kinetic rate parameters, and extensively search for the possibility of a two distinct stable steady states. This procedure itself is very tedious, time consuming and inaccurate.

Here, we develop a systematic approach to reduce the dimensionality of a high dimensional ODE based model of αCaMKII-CPEB1 molecular loop. Our simplification strategy is devised such that the essential features of full scale model are preserved. This simplification process is based on introducing the two key approximations. 1. The synthesis-degradation curve (equation 1) and 2. The fitting function curve (equation 7). Through these approximations the full scale model is reduced to three lower dimensional models. (a) A three dimensional model comprising of equations 6, B-6, and B12. (b) A one dimensional model (equation 6), (c) A one dimensional fourth order polynomial based model (B-18). We can extract the fixed point solution characteristics of this system from all three reduced models however, only reduced model (a) and (b) can provide the dynamical characteristics of this system. Since, during the simplification process it is possible to loose some features therefore, it would be logical to compare the performance of these three reduced models with a full scale model of this loop.

Our results show that the key to performance of three reduced models in locating fixed points of the system is the approximation introduced through equation 1. Since, in this approximation the CaMKII bound in biochemical complexes also degrade along with free CaMKII, which is in contrast to full scale model. Thus, for parametric conditions where the amount of bound CaMKII is negligible the three reduced models yield matching results to full scale model. However, for conditions where there is a non-negligible amount of bound CaMKII the results of three models are not matching with full model. Interestingly, however the results of these three reduced models are matching with each other. Here, this phenomenon of CaMKII trapping in biochemical complexes is simply modeled through an autophosphorylation parameter k_3_. For the k_3 _set at lower values the amount of CaMKII bound is large and thus leading to disagreements between the results from full scale model and reduced models.

The analysis of this paper also provides some intuition on the complex process of polyadenylation based translation. Here, through a systematic simplification process we develop a single variable dynamical equation (eq. 6) which shows that the change in total CaMKII concentration is due to a balance between protein synthesis and degradation. At steady state this equation is composed of two terms i.e., synthesis and degradation term. Thus, the polyadenylation based switching is essentially due to a balance between the synthesis of new proteins and degradation of old ones. If the amount of synthesis is at much faster rate then the degradation rate the balance will shift towards single state high protein concentration, similarly if the degradation rate is much faster then new protein synthesis the balance will tip towards single lower state of switch. So for reversible switching a balance should be maintained between protein synthesis and degradation. This equation also provides an easy method to analyze the effect of other system variables on switching characteristics. For example one parameter which could critically affect the polyadenylation switching is the CPEB1 activation rate through CaMKII i.e., k_5_. For larger value of this parameter the new synthesis rate will increase and at lower value the new synthesis rate will decrease.

In this paper, we simplify the high dimensional ODE model of αCaMKII-CPEB1 loop in such a way that these simplifications preserve the characteristics properties of the full scale ODE model. By reducing the high dimensional model to the minimal plausible scenario, we were able to obtain a single algebraic expression that characterizes the fixed points of these dynamics. We then used these algebraic equations to develop the analytical bifurcation diagrams by perturbing the various parameters (λ and k5). Using this analytical expression we explain how the loss of αCaMKII in synapses due to protein degradation is balanced by synthesis of new αCaMKII molecules. This analysis explains how the pair of two molecules in active and inactive forms and a synthesis based positive feedback loop can lead to a bistable switch. On the basis of these bifurcation diagrams we show that even the simplified version of a polyadenylation based model of αCaMKII can exhibits an up and down state and by perturbing these parameters the system can toggle between an up and a down state. The activity induced increase in total amount of αCaMKII can be maintained for long period of time due to a balance between the new synthesis and degradation of αCaMKII molecule. As we set equation 1 equals to zero the resulting equation 2 describe the steady state value of total amount of αCaMKII. It also shows that total concentration of CaMKII is directly proportional to the amount of CPEB1 phosphorylated and is inversely proportional to the degradation rate. Therefore, when the fraction of CPEB1 phosphorylated is high the amount of CaMKII generated through polyadenylation increases, which will out balance the amount of αCaMKII removed through degradation and there is a net up-regulation of αCaMKII concentration. Similarly this equation also explains that if degradation rate is too fast the compensation provided by new synthesis of αCaMKII molecules will not be enough to balance the loss of CaMKII due to degradation. Our simplification captures the essential properties of protein translation through polyadenylation. It shows that how the activity induced signal converts the inactive form of αCaMKII into active, which is further amplified through an auto-phosphorylation loop. The active and phosphorylated αCaMKII in turn drives the synthesis of a new inactive αCaMKII molecule, through phosphorylating the CPEB1. Here, all these steps are analytically proven as shown by the equations (B1-B9).

Apart, from the approximation introduced through equation 1 another approximation is introduced into this analysis when we tried to develop a single algebraic equation 6 based version of this system. In equation 6 we developed an approximate function XP = g ( XT). In order, to develop a completely accurate solution this function should be analytically developed, however, for this system it was not possible to invert the highly non-linear system of XT as a function of XP. Here, we developed the approximate function XP = g (XT) through a fitting function routine in matlab. The approximate fitting function proved to be very sufficient good in approximating both the upper, lower stable steady state solution and unstable steady state solution., however, it could not accurately approximate the lower stable steady state solution.

The numerical values of different parameters of this molecular loop are described in table 1. Many of these parameters are extracted from previous observations based on experimental and simulation work [16,19,20,25-34]. For example parameters like αCaMKII degradation rates, calcium/calmodulin binding and un-binding rate, rate parameters for αCaMKII auto-phosphorylation loop and rate parameters describing the new synthesis of αCaMKII protein are taken from previous experimental and simulation based observations [27,28,31-33], and [34]. Some other rate parameters such as activation of CPEB1 through phosphorylated αCaMKII are obtained through parameters scaling and matching the observed experimental dynamics. The parameters of table 1 and bifurcation diagrams of Figure 4 and 5 indicate that this molecular system is very robust. For example the k_5 _parameter (describing αCaMKII mediated activation of CPEB1) exhibits the bistable character over a range of three orders of magnitude. This means that even a small activation of CPEB1 through αCaMKII will induce the bistable character to this loop and large strength of αCaMKII activity inhibition is required to reverse the up-state to down. This leads to interesting observation where many of αCaMKII inhibitors are not able to reverse the established L-LTP when applied during the maintenance phase of memory formation [33]. The reduced model of this work and full-scale model [30] can make these predictions.

## Conclusions

This study provides a systematic method to simplify, approximate and analyze a molecular model of polyadenylation loop. This model of polyadenylation loop is based upon de-novo synthesis of new proteins & their activation. The polyadenylation loop operates through a positive feedback loop between a protein and its translation factor. This work shows how to extract low dimensional systems that can be used to obtain analytical solutions for the fixed points of the system and to describe the dynamics of the system. The methods used here have general applicability to the formulation and analysis of many molecular networks.

## Competing interests

The authors declare that they have no competing interests.

## Authors' contributions

NA carried out the modeling, simulation and analysis of this work. HS contributed in conceptual design and polishing of this work. All authors read and approved the final manuscript.

## Supplementary Material

Additional file 1 **Figure S1 The fitting performance of equation7 with original function B-13 (Method B)**. Click here for file
