# Supplementary material for: Regulation of cytoplasmic polyadenylation can generate a bistable switch
Source: BMC Syst Biol. 2012 Feb 15;6:12. doi: 10.1186/1752-0509-6-12 (PMC3359155; doi:10.1186/1752-0509-6-12)

Additional file 1, Figure S1: The fitting performance of equation7 with original function B-13 (Method B)

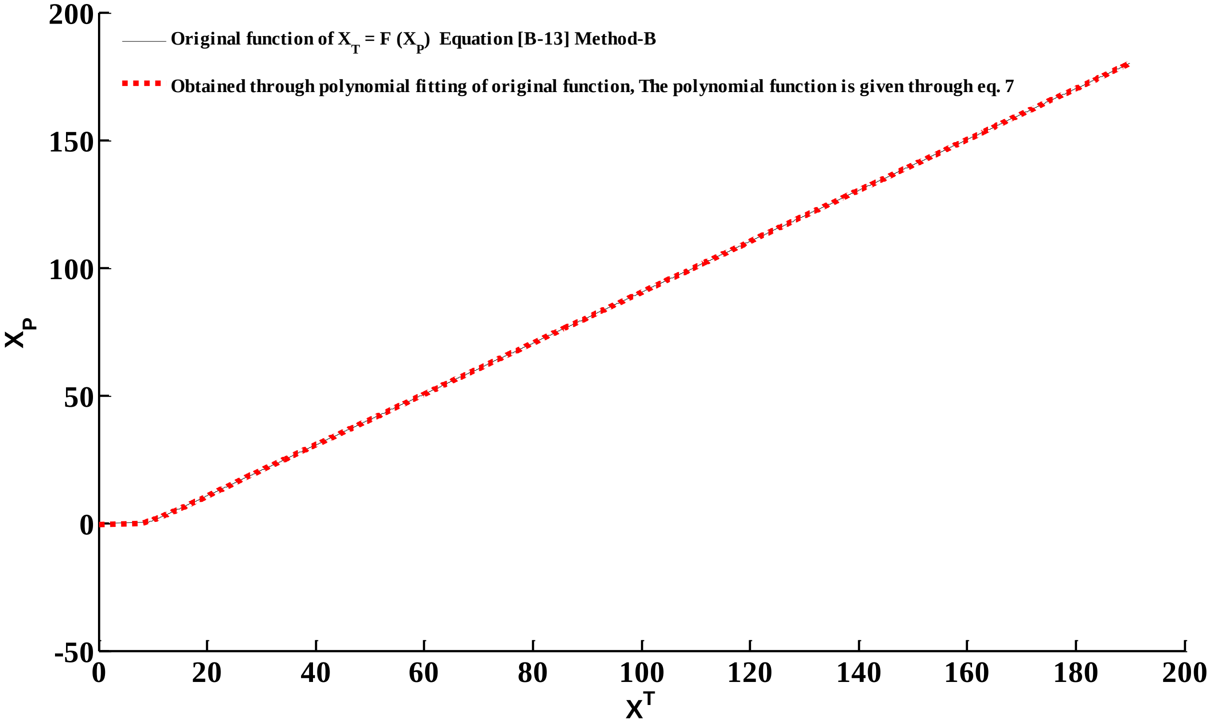

Supplement: Additional file 1 — Figure S1 The fitting performance of equation7 with original function B-13 (Method B). [file 1752-0509-6-12-S1.PDF]
